# Mild Cognitive Impairment and Mild Dementia: The Role of *Ginkgo biloba* (EGb 761^®^)

**DOI:** 10.3390/ph14040305

**Published:** 2021-04-01

**Authors:** Carlo Tomino, Sara Ilari, Vincenzo Solfrizzi, Valentina Malafoglia, Guglielmo Zilio, Patrizia Russo, Stefania Proietti, Federica Marcolongo, Giovanni Scapagnini, Carolina Muscoli, Paolo Maria Rossini

**Affiliations:** 1 Scientific Direction, IRCCS San Raffaele Roma, 00166 Rome, Italy; carlo.tomino@sanraffaele.it (C.T.); Stefania.proietti@sanraffaele.it (S.P.); 2 Department of Health Science, Institute of Research for Food Safety & Health (IRC-FSH), University “Magna Graecia” of Catanzaro, 88201 Catanzaro, Italy; sara.ilari@hotmail.it (S.I.); muscoli@unicz.it (C.M.); 3 Clinica Medica “Frugoni” and Geriatric Medicine-Memory Unit, University of Bari Aldo Moro, 70122 Bari, Italy; vincenzo.solfrizzi@uniba.it; 4 Institute for Research on Pain, ISAL Foundation, Torre Pedrera, 47922 Rimini, Italy; valentinamalafoglia@yahoo.it; 5 Scientific Department, Schwabe Pharma Italia S.r.l., 39044 Egna, Italy; guglielmo.zilio@scwabe.it; 6 Clinical and Molecular Epidemiology, IRCCS San Raffaele Roma, 00166 Rome, Italy; federica.marcolongo@sanraffaele.it; 7 Department of Human Sciences and Quality of Life Promotion, San Raffaele University, Via di Val Cannuta, 247, 00166 Rome, Italy; 8 Department of Medicine and Health Sciences “V. Tiberio”, University of Molise, 86100 Campobasso, Italy; g.scapagnini@gmail.com; 9 Department of Neuroscience & Neurorehabilitation, IRCCS San Raffaele Roma, 00163 Rome, Italy; paolomaria.rossini@sanraffaele.it

**Keywords:** mild cognitive impairment (MCI), mild dementia, Alzheimer’s disease, Ginkgo biloba (Egb761^®^), Tebonin, anti-dementia drugs, randomized controlled trials

## Abstract

Mild cognitive impairment (MCI) and dementia are clinically prevalent in the elderly. There is a high risk of cognitive decline in patients diagnosed with MCI or dementia. This review describes the effectiveness of Ginkgo biloba leaf special extract EGb 761^®^ for the treatment of dementia syndromes and EGb 761^®^ combination therapy with other medications for symptomatic dementia. This drug has shown convincing results, improving cognitive function, neuropsychiatric symptoms and consequent reduction of caregiver stress and maintenance of autonomy in patients with age-related cognitive decline, MCI and mild to moderate dementia. Currently, there is little evidence to support the combination therapy with anti-dementia drugs and, therefore, more evidence is needed to evaluate the role of EGb 761^®^ in mixed therapy.

## 1. Introduction

Mild cognitive impairment (MCI) is a clinically relevant health problem in the elderly and it is considered an intermediate state between normal aging and dementia [1]. This state can progress to dementia, and Alzheimer’s disease (AD) is the most common form of neurodegenerative disorder [2]. The prevalence of MCI in the population over 60 is approximately 5.9%, it tends to increase with age, and is more common in men. The MCI development varies according to numerous risk factors that, beyond age, also include genetics, comorbidities, chronic diseases as vascular risk factors, pulmonary diseases, depression, metabolic risk such as diabetes mellitus, hypertension and also tobacco utilization [3].

Currently, despite pharmacological new findings, there is still no specific drug approved by the Food and Drug Administration (FDA) for the treatment of MCI. The only drugs used, approved by the FDA for the treatment of mild and moderate AD, are Acetylcholinesterase (AChE) inhibitors: AChEIs or memantines, although their effects are not very effective and have numerous side effects such as nausea, bradycardia, fatigue [4]. Moreover, non-pharmacological treatments such as behavioral interventions, psychosocial support, physical activity including rehabilitation programs, diet and cognitive stimulation shown a benefit to patients. [4,5].

Additionally, an important factor involved in the etiology of cognitive decline is inflammation and the subsequent development of oxidative stress.

Consequently, several studies suggested an important role of diet in the prevention of neurodegenerative diseases, showing a protective role against the damaging effects of neuroinflammation and oxidative stress [4,5].

Indeed, the Mediterranean diet has been shown to reduce the incidence of mild cognitive impairment (MCI) and, possibly, the conversion of MCI to dementia [6]. Vitamins, minerals, polyphenols have been associated to the prevention of cognitive impairment due to their antioxidant effects, such as the reduction of free radical species and therefore the oxidative stress development [7,8,9,10].

Considering polyphenols deriving from plants, *Ginkgo biloba* is the oldest living tree species in the world and is one of the most studied herbals for cognitive disorders and AD [11,12].

In traditional medicine Ginkgo leaves were used mainly for the treatment of respiratory and cardiovascular disorders while in Chinese medicine, *Ginkgo biloba* seeds were used to treat pulmonary symptoms, alcohol abuse and bladder infections [13]. The modern use of *Ginkgo biloba* extract is all about leaf-based preparations and numerous health benefits have been attributed to its utilization. Recent studies showed an important role of *Ginkgo biloba* in cognitive improvement [14].

In particular, it has been observed that *Ginkgo biloba* extract EGb 761^®^ could play protective effect roles and it is an effective treatment in both Alzheimer’s diseases and vascular dementia [15]. Its pharmacological effect is based on antioxidant, anti-inflammatory and anti-apoptotic action and the defense against mitochondrial dysfunction [16]. These mechanisms are considered to contribute to cognitive improvement, impeding the evolvement of neurodegenerative diseases.

Indeed, vegetal origin drug based on EGb 761^®^ active principle, registered as “well established use” is authorized in many European States and it is considered the only drug treatment recommended in the guidelines for the treatment of MCI.

This review describes the main clinical and preclinical human studies highlighting the efficacy of the *Ginkgo biloba* EGb 761^®^ for the treatment of MCI and dementia syndrome, and EGb 761^®^ combination therapy with other symptomatic drugs for dementia.

### 1.1. Mild Cognitive Impairment (MCI) and Mild Dementia: Recent Classification Criteria, Pathogenesis, Therapeutic Aspects

Neurodegenerative diseases involve different groups of pathologies of the central nervous system, characterized by a progressive loss of synaptic transmission in some specific neuronal circuits. This information flow deficit, without any clear structural damage, is then followed by progressive neuronal death with loss of volume and obvious accumulation of toxic substances in both extra- (e.g., amyloid beta plaques) and intra-neuronal spaces (e.g., neurofibrillary tangles) [17,18,19]. Moreover, these disorders are classified considering clinical features (Parkinson or dementia), anatomic distribution of neurodegeneration (frontotemporal degenerations, spinocerebellar degenerations), or as a result of molecular abnormalities [20]. Depending on the type of disease, neuronal deterioration can result in cognitive deficits, dementia, motor alterations, behavioral and psychological disorders [21].

**Figure 1:**
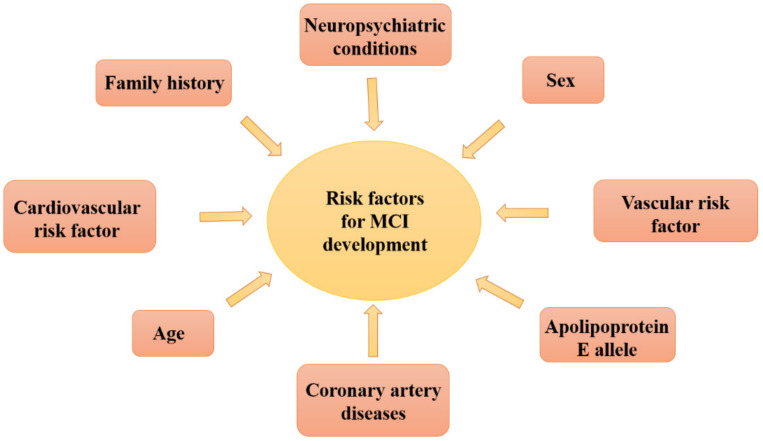
Risk factors for MCI development.

Specifically, mild cognitive impairment (MCI) and dementia are symptoms of various neurodegenerative diseases characterized by cognitive decline, the most common are Alzheimer’s disease (AD), vascular dementia and Lewy Body’s disease [22]. Unlike MCI, in mild dementia the interference with daily life is evident [2]. In recent years, MCI prevalence in the over 60 population is estimated to be around 6–22% [23], depending on the method of evaluation, the population examined and other factors. MCI is clinically heterogeneous and different factors can increase the risk of MCI development (Figure 1).

Diagnostic criteria employed to identify MCI include a decrease in the performance of several cognitive functions, in one or more domains, related to memory, orientation or verbal skills [17,18]. This cognitive decline, which is still common in the elderly population, is not necessarily indicative of incipient dementia.

**Figure 2:**
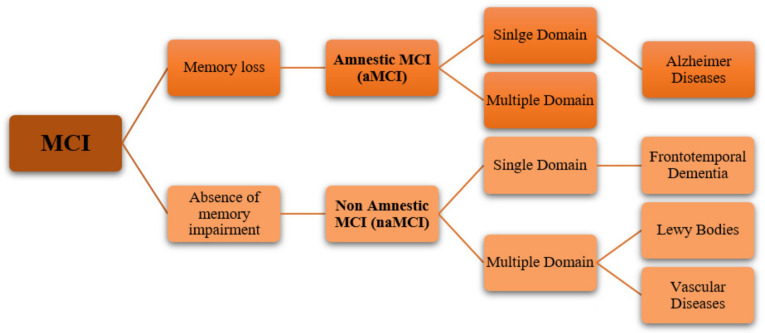
Schematic representation of MCI subtypes.

A MCI classification has been proposed to distinguish between amnestic MCI (aMCI, where memory is significantly impaired) and non-amnestic MCI (naMCI, where memory remains intact). Furthermore, MCI may involve impairment in a single cognitive domain or in multiple cognitive domains (Figure 2). The combination of the several clinical subtype with degenerative, psychiatric or vascular etiology could be an important predicting factor of the type of dementia that the MCI patient would develop (AD, vascular dementia, frontotemporal dementia, Lewy bodies) [24]. The progression of different MCI subtypes into a particular type of dementia has not yet been well understood [25].

Therefore, patient’s history is critical to recognize and detect various clinical signs and making a diagnosis.

Cognitive decline in the elderly is a common problem and healthcare providers are often the first point of contact for patients and their families. Generally only elderly with moderate to severe dementia are subject to medical treatment, while MCI patients are often not even diagnosed by primary care providers [26].

MCI has attracted a lot of interest within the scientific community because it represents a stage between normal age-related cognitive function and a clinically probable diagnosis of several dementias [27]. It has become the “target” of many recent clinical trials with potentially disease-modifying drugs.

One of the scientific and public health problems is the fact that neuropsychological tests do not distinguish MCI patients who will never develop dementia from those who are in a prodromal-to-dementia condition [22].

Currently, medical history and mental health examination are the most commonly used tools to diagnose MCI or mild dementia [28]. Specifically, through medical history, the clinician determines whether or not there is a decline in the patient’s daily functions while, through the mental health examination, the clinician establishes a patient’s noticeable cognitive impairment [28]. Another important tool that could be performed for MCI and dementia diagnosis is the general neurological examination whose role in the diagnostic process is to understand the etiology of cognitive disorder and to exclude/confirm the presence of modifiable factors (i.e., subdural hematoma, frontal lobe neoplasm, dysmetabolic syndromes, etc.) [26]. Consequently, over the last twenty years, several attempts have been made to draw up general investigations for this disorder [29,30].

Therefore, after a comparison with normal subjects of the same age, a greater degree of memory impairment has been noted in MCI patients with little or absent involvement of common activities of daily living [31]. As the disease progresses, a sub-sequential loss of cognitive function, a loss of functional independence, and the development of behavioral problems occur. Therefore, early diagnosis and treatment may delay the progression of symptoms.

For this reason, the concept of “biomarkers” is now emerging, as instrumental examinations that, in association with neuropsychological tests, could be used to identify individuals with MCI who are already in a prodromal-to-dementia stage [22]. Due to the general interest in this area, the Ministry of Health and Italian Drug Agency (AIFA) has designed the INTERCEPTOR project that aims to compare 6 different biomarkers (Fluorodeoxyglucose (FDG)-Positron Emission Tomography (PET), neuropsychological tests, liquor for beta and tau, electroencephalogram (EEG) for connectivity through graphs, magnetic resonance imaging (MRI) for hippocampal volumetry, genetics for Apolipoprotein E (ApoE)) and an organizational model to validate a sustainable, non-invasive and widely widespread method across the country for the diagnosis of prodromal MCI. This will be important to identify high-risk individuals, to find the resources for early pharmacological and non-pharmacological-treatments, as well as for the delivery of any disease-modifying drugs, which should be effective among several trials.

### 1.2. Therapeutic Approaches in Mild Cognitive Impairment and Mild Dementia

Currently, only pharmacological treatments with modest value are approved for mild dementia due to AD, while none have been approved for MCI in Italy. In fact, despite numerous randomized clinical trials (RCTs) being conducted in MCI patients, none have been able to demonstrate the effectiveness at delaying disease progression [30,32]. In addition, it is important to note that (when symptoms are disabling) the delaying or slowing down the onset or the worsening of dementia-related symptoms can have a major impact on the social and health costs. An extension of the total/partial autonomy time can reduce the total costs of the disease by about 50% [33].

To date, the only drugs used to treat symptoms are cholinesterase inhibitors and memantines.

Indeed, several studies identified a cholinergic deficit in subjects with dementia [34]. By Positron Emission Tomography (PET) and Magnetic Resonance Imaging (MRI), a reduction in AChE activity and an atrophy of the nucleus basalis of Meynert (the main source of AChE, the origin of cholinergic neurotransmission and projections to the cortical brain areas associated with learning and memory) was shown. Therefore, actual therapeutic hypothesis in AD is to restore physiological levels of acetylcholine through the inhibition of acetylcholinesterase enzyme activity [35]. Reversible AChEIs drugs are available; they are lipophilic enough to overcome the blood–brain barrier to act preferably on the central nervous system [36].

Among these drugs, the most studied are donepezil, physostigmine (no longer in use), rivastigmine and galantamine, approved by FDA for the treatment of mild dementia due to AD, but no treatment has yet been approved by the US FDA for MCI.

Another important therapeutic approach involves the use of drugs that act directly on the glutamatergic system, such as memantine [35]. In particular, memantine is a noncompetitive N-methyl-D-aspartate receptor (NMDA) receptors-antagonist and provides symptomatic treatment of dementia inhibiting the pathological activation of NMDA receptors [37]. Its neuroprotective effects have been demonstrated in several neurological disorders. Indeed, studies conducted in ischemic models showed that an increase in NMDA receptor antagonist drugs, in the blood, led to a decrease in glucose metabolism, thus supporting the memantine neuroprotective effect.

Memantine is used for cognitive disorders in patients with moderate to severe AD. No drugs have been approved for the prevention of these disorders [34].

For this reason, the focus has shifted towards non-pharmacological treatments that involve behavioral interventions, psychosocial support and cognitive training. Such measures are usually supplemented with drug treatment, and their positive effectiveness in the overall clinical patient’s management have been demonstrated [22].

Cognitive training (of different types, and with different functional goals: Reality-Orientation Therapy, Validation Therapy, Reminiscence Therapy, various cognitive stimulation therapy programs—Cognitive Stimulation Therapy, etc.) showed results both in stimulating and reinforcing neuro-cognitive abilities, as well as in improving the execution of daily life tasks [22].

The effect of moderate physical and motor activity, especially in the intermediate stages of the disease, seems to be positive for the tone of mood, the physical wellness- and the regularization of behavioral disorders, sleep and nutrition [22].

Furthermore, inflammation and therefore oxidative stress are important factors involved in cognitive decline [14]. Particularly, oxidative stress appears to be involved in the early phase of AD and MCI and, therefore, could be considered as a prodromal phase of dementia. For this reason, various antioxidant therapies have been shown to influence the onset and progression of AD [38].

Natural polyphenolic compounds confer an antioxidant effect thus reducing the development of free radicals, restoring the endogenous antioxidant defense and carrying out important neuroprotective effects for the body [14].

### 1.3. Ginkgo Biloba: From Traditional Chinese Medicine to Anti-Dementia Drug Based on Scientific Evidence

*Ginkgo biloba* is one of the oldest tree species in the planet used especially for its health properties [13].

Ginkgo and derived pharmaceutical formulations have a tradition in the Chinese medicine; at the beginning seeds and then, in the modern phytotherapy, leaves extracts were used [13,39].

In the 1960s, Dr Willmar Schwabe pharmaceuticals introduced a drug based on leaf extract that contained terpenoids and flavonoid glycosides and organic acids. Several studies, conducted in cell and animal models, showed the neuroprotective effects of this drug.

Later, this product has been modified to improve the good characteristics and decrease side effects; in the 1980s, it wasproposed with the name of EGb 761^®^ with an enrichment in flavonoids, ginkgolides and bilobalide, and a reduction of ginkgolic acids [39].

Herbal medicinal products with Ginkgo biloba leaf extracts such as EGb 761^®^ active principle, belonging to the cognitive drug category (*Ginkgo biloba*, ATC cod: NO6DX02) authorized in many European States.

*Ginkgo biloba L.* (Family: *Ginkgoaceae*) dry leaves are used to satisfy the European pharmacopeia and specific pharmaceutical companies requisites. Tebonin indication is approved by EMA report and monograph, as a vegetal drug for the improvement of cognitive deterioration (linked to age) and life quality in the mild dementia.

**Figure 3:**
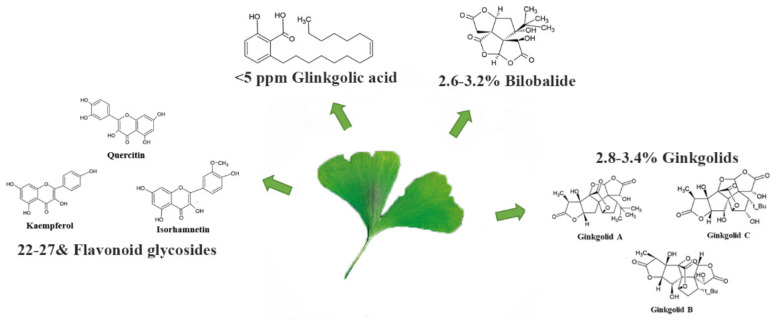
EGb 761^®^ active components.

Particularly, EGb 761^®^ is a light yellow–brown/yellow–orange bitter powder that has to satisfy all the requisites of European pharmacopeia actual monograph “Ginkgo dry extract, refined and quantified”. The flavonoid fraction is responsible for the antioxidant properties of EGb 761^®^. Particularly, EGb 761^®^ contains between the 22 and 27% flavonoid glycosides (i.e., kaempferol, quercetin and isorhamnetin) that inhibit oxidation of tert-butylhydroperoxide [40], 2.8–3.4% ginkgolids A, B and C, 2.6–3.2% bilobalide and contains less than 5 ppm ginkgolic acids (Figure 3). The Ginkgo flavonoids and the terpene lactones (ginkgolide A, B, and C diterpenes and the sesquiterpene bilobalide, all provided with three lactone rings) are EGb 761^®^ ingredients with therapeutic characteristics (Figure 3). In particular, ginkgolide A has been demonstrated to lack the ability to scavenge the superoxide, while the superoxide scavenging activity of bilobalide and the ginkgolides B, C is still not sure [41].

From the beginning, EGb 761^®^ has been used for peripheral and central vascular disorder diseases [42]. Currently, EGb 761^®^ utilization has been abandoned in the most peripheral vascular diseases, as well as Raynaud syndrome, the intermitted claudication and peripheral arteriopathy, despite the beneficial evidences obtained in placebo-controlled trials [42,43]. Positive effects have been observed also for cerebrovascular diseases, above all for vascular cognitive impairment.

Later, it was also used for cognitive impairment associated with aging, demonstrating beneficial in different clinical trials, in line with the international diagnostic criteria modification but with several limitation about the most updated guidelines for Alzheimer’s disease drugs development [6,13,44,45,46].

In Germany, the E commission has approved the monograph which defines the use of *Ginkgo biloba* undefined leaves preparation, for cerebral and arterial blood circulation, for vertigo and the reinforcement of the vascular system, (i.e., veins), for the stimulation of blood circulation (i.e., after psychotropic and neurotropic therapy). The specific monograph of the purified and titrated product, extracted from *Ginkgo biloba* leaf (DER 35-67:1), described its use in the symptomatic treatment of cerebral insufficiency, as a part of a general therapeutic strategy for dementia with the following principal symptoms: deficit of memory, concentration and mood disturbance, vertigo, tinnitus and headache [47,48].

The main target group includes primary degenerative dementia and/or vascular dementia patients. Moreover, it has been used to improve the free from pain motor skills in patients with second stage of peripheral occluding arteriopathy, according to Fontaine classification.

The 2003 European Scientific Cooperative On Phytotherapy (ESCOP) monograph [49] describes the *Ginkgo biloba* leaf utilization for the symptomatic treatment of moderate and mild dementia, involving degenerative primary dementia, vascular dementia, mixed forms and cerebral impairment; for neurosensorial impediments as well as dizziness/vertigo and tinnitus; for cognitive performance improvement; for the symptomatic treatment of occlusive peripheral arteries disease.

The 2006 British Herbal Compendium [50] lists the following indications for *Ginkgo biloba* leaves: symptomatic treatment of moderate and mild dementia, including primary degenerative dementia, such as Alzheimer’s, multi-infarct dementia and mixed forms; treatment of symptoms of cerebral vascular impairment and concentration/memory problems, confusion, deficit of energy and initiative, anxiety and depression; improvement of cognitive performances; neuro-sensorial disturbance as vertigo and visual dysfunction.

#### 1.3.1. EGb 761^®^ in Basic Research: The Mechanism of Action

EGb 761^®^ pharmacological effect is based on four main neurobiological mechanisms: 1. increase in neurogenesis and synaptogenesis, 2. mitochondrial DNA oxidation prevention, followed by stabilization of mitochondrial membranes which slows down aging, 3. neuro-transmission improvement and 4. improved microcirculation.

**Figure 4:**
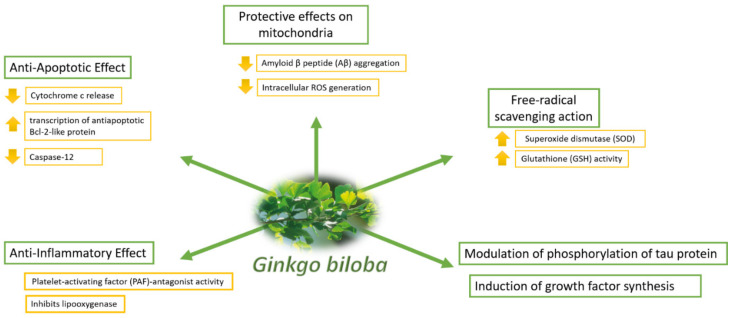
Mechanisms of action of *Ginkgo biloba* [41].

Figure 4 shows the possible mechanisms of action.

The different extract active components act simultaneously, in the cerebral context, with different mechanisms to enhance beneficial effects on cognitive functions and improve neuro-protection. Several preclinical experimental studies showed that EGb 761^®^ has antioxidant proprieties due to its flavonoids [51,52,53]. These components protect against several neurotoxic agents and in a specific manner against the neurotoxicity from amyloid beta oligomers [47]. The antioxidant action can be explicated by a direct reactive oxygen species (ROS) scavenging or through the modulation of specific mechanisms of signaling and transcription factors, able to stimulate the cellular repair system and to amplify the endogenous antioxidant defenses [48,54,55].

A peculiar EGb 761^®^ activity is to improve mitochondrial dysfunction during the aging and in cerebral cognitive impairment [53,56,57,58,59,60]. This mechanism underlines EGb 761^®^ neuroprotection. Thus, several studies demonstrated that functional mitochondrial homeostasis plays a critical role in neuro degeneration and the cognitive deterioration associated with dementia [48,61].

EGb 761^®^ reduces mitochondrial ROS production and protects mitochondria and mitochondrial complexes of the respiratory chain from ROS, it increases the energetic metabolism and ATP availability [53,57,62,63,64,65].

Preclinical studies demonstrated that EGb 761^®^ also has a positive role in neuroinflammation [66,67], including a specific inhibitory action of inflammatory molecules transcription factors at neuronal level and in the microglia [68]. Moreover, EGb 761^®^ can inhibit the aggregation and production of amyloid beta [66,69,70,71,72,73,74,75,76,77]. Recently, it has been observed that EGb 761^®^, and in particular its ginkgolid A (but not ginkgolid B and C), and flavonoid components, enhanced autophagic activity and degradation of phosphorylated TAU protein in lysosomes and neuron [78]. Moreover, EGb 761^®^ stimulated the dopaminergic and noradrenergic neurotransmission [79,80], thus improving cognitive functions in the elderly [81].

Cellular and animal studies demonstrated that EGb 761^®^ helps also synaptic functions and the neuronal plasticity, including the neuritogenesis, the spinal cord density, the long-term potentiation, and neurogenesis. These effects are stronger in experimental models of hypoglycemia, hypoxia, amyloid beta exposition, oxidative stress [58,82,83].

#### 1.3.2. EGb 761^®^ in Preclinical and Clinical Dementia: Evidence of Efficacy

Although it has been used in the treatment of neuro-cognitive disorders for several years, the expert evaluation of EGb 761^®^ has become unanimously positive over the last years [15,44]; however, in the last few years, scientific reviews and meta-analysis studies results have been pivotal in the achievement of EGb 761^®^ as a drug for dementia, as underlined in the guidelines of cognitive disorders treatments. Table 1 describes the different types of dementia and the types of pharmacological treatments currently in use [84,85,86,87,88,89].

#### 1.3.3. EGb 761^®^ in vMCI and Dementia Prevention

Prospective observational studies, such as the clinical study conducted by Amieva et al., 2013 [90] which involved 3612 patients, aged over 65, from the South of France, highlighted a slower progression of cognitive impairment in the EGb 761^®^ group of patients than in Piracetam ones. This difference has been confirmed also through a multiple-choice visual memory test (Benton Visual Retention Test (BVRT)) and language skills test (Isaacs Set Test (IST)). Moreover, EGb 761^®^ patients showed a significant reduction of psychotropic drug consumption, considering that, in dementia experimental clinical trials, ADAS-Cog is the more used formulation (Table 2).

A clinical study, conducted by Grass-Kapanke et al., in 2011 [91], observed EGb 761^®^ positive effects with very mild cognitive impairment (vMCI). In this study, 300 vMCI patients, aged 45 to 65 years, received EGb 761^®^ (240 mg/die) or placebo, for 12 weeks. These patients showed an improvement of memory performances, measured through Wechsler Memory Scale III (human face recognition in pictures) and a significant attention improvement, by using the Vienna Test System Work Performance Series (a computerized math test to keep concentration) (Table 2).

A randomized, double blind, placebo controlled, multicentric study (GuidAge), including French people with subjective memory deficit and enrolled for primary clinical care for Alzheimer’s evaluation, prospective followed for more than 5 years [92], showed that EGb 761^®^ treatment could not prevent dementia incidence.

A post hoc analysis of a subgroup of people on going EGb 761^®^ for at least 4 years, showed a significant reduction of dementia development (50% more than placebo group). This long-term effect made scientists doubt and confirm the failure of the statistical models used [93].

In conclusion, these studies affirm a positive effect of Ginkgo biloba in delaying cognitive impairment and improving memory performance in people with vMCI. Currently, the question is still open whether EGb 761^®^ could potentially prevent dementia yet.

### 1.4. EGb 761^®^ in MCI

Gavrilova and colleagues, in 2014 [94], evaluated EGb 761^®^ (240 mg/die) versus placebo for 24 weeks in 160 patients with amnesic MCI (age > 55 years old). All the neuropsychiatric symptoms, measured through Neuropsychiatric Inventory (NPI), improved in patients treated with EGb 761^®^. They reached a better diagnosis also for anxiety, depression and visual-motor and cognitive aspect (Table 2).

#### 1.4.1. EGb 761^®^ Efficacy in Mild and Moderate Dementia with or without Neuropsychiatric Disorders

The older controlled clinical trials using EGb 761^®^ versus placebo, have demonstrated a moderate improvement of cognitive functions and activities of daily living (ADL).

In the last 10 years, randomized and controlled trials and sub sequential meta-analysis, considering people affected by AD and MCI associated with neuro-psychiatric symptoms (NPS), have renewed interest on EGb 761^®^ benefits at the dosage of 240 mg/die.

In particular, neuro-psychiatric symptoms, known as “behavioral and psychological dementia symptoms (BPSD)” represent various not-cognitive symptoms groups and behavioral attitude of dementia patients, which dramatically increase during the development of pathology, compromising also “caregivers” life quality. In the last years, EGb 761^®^ has been largely studied in BPSD; in addition to neuro psychiatric symptoms improvement, also the recovery of behavioral ability enhancing has been detected. Indeed, three EGb 761^®^ studies [95,96,97] have demonstrated neuro psychiatric improvement for Neuropsychiatric Inventory (NPI) symptoms not only for Alzheimer’s but also on vascular dementia and mixed forms. AD and Vascular Dementia (VaD) have been included in these studies, where the Caregiver Distress Score was improved in patients on going EGb 761^®^, by showing a stress reduction for patient’s relatives (Table 2) [90,91,92,93,94,95,96,97].

EGb 761^®^ in people affected by MCI associated with NPS was also studied, observing an improvement in NPS and in cognitive performance in EGb 761^®^ patients [94].

**Table 2:** Clinical studies on EGb 761^®^ in patients with MCI, vMCI, and dementia.

Inclusion Criteria	Treatment Groups	Results	References
**EGb 761^®^ in vMCI, MCI and dementia prevention**			
Non-demented patients	EGb 761^®^ or Piracetam and placebo, data collected on cognitive function over a period of twenty years	Patients treated with EGb 761^®^ highlighted a slower cognitive impairment than in Piracetam group. Moreover, EGb 761^®^ patients showed a significant reduction of psychotropic drugs assumption.	[90]
Patients with very mild cognitive impairment and low functioning	EGb 761^®^ (240 mg/die) or placebo, for 12 weeks	Patients treated with EGb 761^®^ showed an improvement of memory performances, measured through Wechsler Memory Scale III (human face recognition in pictures) and a significant attention improvement, by using the Vienna Test System Work Performance Series (a computerized math test to keep concentration).	[91]
**EGb 761^®^ MCI, neurocognitive deficit and dementia**			
Patients with amnesic MCI	EGb 761^®^ (240 mg/die) or placebo, for 24 weeks	Patients treated with EGb 761^®^ showed improvement in all the neuropsychiatric symptoms, measured through Neuropsychiatrics Inventory (NPI) sympoms.	[94]
Patients with normal cognitive function or MCI	EGb 761^®^ (240 mg/die) or placebo, patients were evaluated every 6 months	This study didn’t demonstrate any significant benefit to prevent dementia development with EGb 761^®^ treatment versus placebo.	[95]
Outpatients with mild to moderate dementia (AD or VaD)	EGb 761^®^ (240 mg/die) or placebo, for 24 weeks	Patients treated with EGb 761^®^ demonstrated neuro psychiatric improvement for Neuropsychiatric Inventory (NPI) symptoms.	[96]
Outpatients 24-week with mild to moderate dementia (Alzheimer’s disease or vascular dementia) associated with neuropsychiatric symptoms	EGb 761^®^ (240 mg/die) or placebo, for 24 weeks	Treatment with EGb 761^®^ led to a significant and clinically relevant improvement in patients’ cognition, psychopathology, functional measures and quality of life.	[97]

#### 1.4.2. EGb 761^®^ Efficacy and Combined (AChEIs and EGb 761^®^ Association) or Compared (AChEIs versus EGb 761^®^) Therapy in Mild or Moderate Dementia

**Figure 5:**
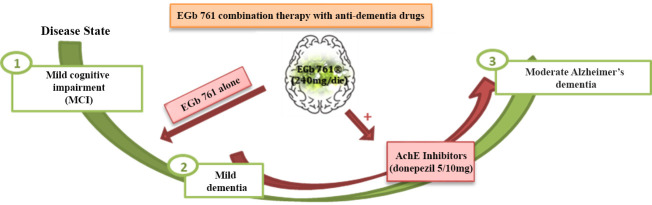
EGb 761^®^ in association with anti-dementia drugs.

EGb 761^®^ in association with anti-dementia drugs have been studied. The first result derived from GINDON study [98], which analyzed the potential benefit of a combined therapy with EGb 761^®^ and donepezil, after 22 weeks in 96 Alzheimer’s patients with neuro psychiatric disorders (Figure 5).

This study has showed a moderate but not significant benefit at the EGb 761^®^ 240 mg/die + donepezil 5/10 mg/die dosage, in monotherapy, versus the single treatment with EGb 761^®^ 240 mg/die or the single treatment with donepezil 5/10 mg/die, considering cognitive, physic and functional outcomes and neuro psychiatric disorders (Figure 5).

Further evidence regarding EGb 761^®^ and AChEIs combination cognitive performances benefits derive from a prospective ICTUS study, involving 828 patients with mild and moderate Alzheimer’s, for 1 year [99]. These patients were treated with donepezil (55%), rivastigmin (27%) or galantamin (18%) with or without co-administration of EGb 761^®^ (120 mg/die). After 12 months, patients ongoing also EGb 761^®^ showed better results of Mini Mental State Examination (MMSE) than patients using only AChEIs (+1.9 points on MMSE, *p* = 0.005).

However, recent studies observed a similar effect among patients with AD who received EGb 761^®^ or AChEI.

In particular, Rapp and colleagues, in 2018 [100], evaluated the efficacy of EGb 761^®^ (240 mg/die) or donepezil (5/10 mg) in 189 patients with Alzheimer’s disease (≥age 80 years old). Similar effects on cognitive symptoms, measured by MMSE over 12 months, resulting from the use of EGb 761^®^ and donepezil in patients with AD were observed in this study.

Similarly, Mazza et al., in 2006, conducted a study on 76 patients (aged 50 to 80 years) with mild to moderate dementia, who received *Ginkgo biloba* (160 mg/die), donepezil (5 mg/die) or placebo for 24-week [101].

Results of this study also showed no differences in the efficacy of EGb 761^®^ and donepezil in the treatment of mild to moderate Alzheimer’s dementia.

#### 1.4.3. EGb 761^®^ Efficacy Verified through RCTs Meta-Analysis in MCI, Mild and Moderate Dementia with or without Behavior Deficits

In the last 10 years, meta-analysis and RCTs results on patients affected by dementia, associated with psycho-behavioral deficits, have led to a new interest for EGb 761^®^. Tan and colleagues [44] estimated EGb 761^®^ effect on 2561 patients with cognitive deficit and dementia (9 RCTs) after 22–26 weeks (Table 3) [15,44].

The results of this work demonstrated EGb 761^®^ benefits on cognitive decline stabilization or slowing down, on ADL and neuro psychiatric deficit for MCI, Alzheimer’s and dementia (with/without neuro psychiatric problems) patients. Other analysis in the same work showed differences in the efficacy of EGb 761^®^ different dosages, underling the best treatment with 240 mg/die. Tan and colleagues, 2015, [44], as in the case of Cochrane meta-analysis [102,103], considered only randomized studies of patients with cognitive function compromised or dementia, with the same EGb 761^®^ dosage and similar follow up timing, with the only difference in the selection of the best trials (better dementia diagnosis). Moreover, Tan and coworkers identified and included three RCTs published in that period [94,96,97] and showing an EGb 761^®^ benefit at the dosage of 240 mg/die, for dementia, AD and MCI associated with NPS. After that, a Gauthier and Schlaefke, 2014, [15] meta-analysis confirmed EGb 761^®^ efficacy and tolerability in patients with dementia (Table 3).

This is a well-designed meta-analysis, involving good quality placebo-controlled studies with at least 200 randomized patients. EGb 761^®^ efficacy has been demonstrated on cognitive functions (*p* = 0.03), ADL (*p* < 0.001) and on the clinical impression scales (*p* = 0.01).

The best result in term of EGb 761^®^ best dosage on cognitive functions has been obtained with 240 mg/die, in studies including significant neuro psychiatric deficits patients. In the seven selected studies of this meta-analysis, four of them enrolled only patients with significative neuro psychiatric symptoms, two of them patients with neuro psychiatric symptoms, and one study excluded patients with significant neuro psychiatric deficits.

A 2015 meta-analysis [104], demonstrated EGb 761^®^ benefits versus placebo for cognitive deficits, through Syndrom–Kurztest [SKT] analysis, in AD and AD + VaD patients (Table 3) [15,44,104,105,106,107].

Another meta-analysis on patient with dementia and neuropsychiatric deficits [105] showed that 240 mg/die of EGb 761^®^ significantly improved the cognitive functions, NPS, caregiver stress associated with NPS, ADL and clinical impression, versus placebo in AD, VaD and AD+ Cardio Vascular Disease (CVD) (*p* < 0.001 for all the analysis) out patients.

A 2016 meta-analysis [106] showed an important activity of Ginkgo extract in improving cognition, behavior and activities of daily living in MCI and dementia. One of the most important outcomes was that the efficacy was dose-dependent and only convincing with a daily dose of 240 mg. Its utilization was safe and the adverse events (AEs) were at placebo level. In the sub-group of Alzheimer’s patients, less AEs arose compared to placebo. Cases of dizziness, Angina pectoris and headache were also less frequent in the active substance group.

Zhang 2016’s publication [106] is the result of an analysis of ten systematic reviews and meta-analyses, in which the efficacy of Ginkgo special extract for cognitive disorders was assessed (Table 3) [15,44,104,105,106,107].

At the end, in a recent meta-analysis of Savaskan and colleagues, in 2018 [107], it has been demonstrated that EGb 761^®^ versus placebo is more efficient for neuro psychiatric symptoms, measured through NPI in AD, VaD or AD + CVD patients, except for delirium, hallucination and euphoria (Table 3) [15,44,104,105,106,107].

Moreover, EGb 761^®^ reduced also the risk of new neuro psychiatric symptoms onset, as the caregiver stress.

**Table 3:** Meta-analysis studies on the efficacy of EGb 761^®^ in patients with MCI, mild and moderate dementia with or without behavioral deficits.

Meta-Analysis Studies (Inclusion Criteria)	Treatment Groups	Results	References
Patients affected by dementia, associated with psycho-behavioral deficits	EGb 761^®^ (240 mg/die) or placebo	Patients treated with EGb 761^®^ showed benefits on cognitive decline stabilization or slowing down, on ADL and neuro psychiatric deficit for MCI, Alzheimer’s and dementia (with/without neuropsychiatric problems) patients.	[44]
Patients with a diagnosis of AD, VaD, or mixed dementia	EGb 761^®^ (120 mg/die) or EGb 761^®^ (240 mg/die) or placebo	EGb 761^®^ (240 mg/die; best dosage) has been demonstrated efficacy on cognitive functions, including significant neuropsychiatric deficits in patients with dementia.	[15]
Patients with AD or AD + VaD patients	EGb 761^®^ (120 mg/die) or EG b761^®^ (240 mg/die) or placebo	Patients treated with EGb 761^®^ (240 mg/die) showed benefits versus placebo for cognitive deficits, through Syndrom–Kurztest [SKT] analysis.	[104]
Patients with the diagnosis of AD, VaD or mixed dementia with behavioral and psychological symptoms (BPSD)	EGb 761^®^ (240 mg/die) or placebo	EGb 761^®^ ginkgo biloba extract (240 mg/die) improved the patients’ cognitive performance, BPSD, functional abilities and general condition.	[105]
Patients with MCI and dementia (AD, VaD or AD + VaD, mixed dementia)	EGb 761^®^ (240 mg/die) or placebo	EGb 761^®^ showed benefit versus placebo for cognition, behavior and activities of daily living in patients with MCI and dementia.	[106]
Patients with dementia (probable AD, VaD or AD +CVD)	EGb 761^®^ (240 mg/die) or placebo	EGb 761^®^ improved neuro-psychiatric symptoms, in AD, VaD or AD + CVD patients, except for delirium, hallucination and euphoria.	[107]

## 2. Discussion and Conclusions

MCI and mild dementia are important diseases affecting our society and, currently, no drug treatment for MCI has been approved in Italy [108]. Specifically, MCI represents an intriguing clinical entity and, until now, the only clinically diagnosed pre-dementia stage.

This review describes the efficacy of the *Ginkgo biloba* leaves extract in the treatment of MCI and dementia syndrome [30].

The published evidence investigating the association between EGb 761^®^ 240 mg/die and placebo for cognitive deficits were reviewed and a meta-analysis was performed.

**Figure 6:**
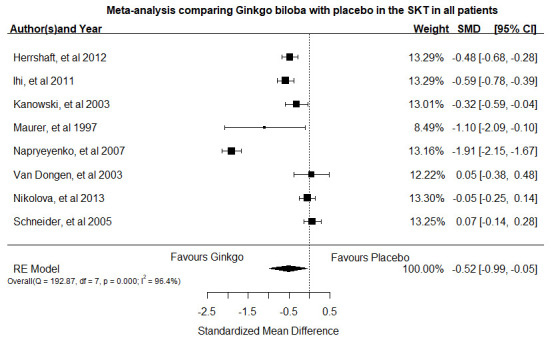
Forest plot of meta-analysis for EGb 761^®^ 240 mg/die and placebo using random-effect model. Meta-analysis of the data obtained from the systematic literature review was conducted according to the guidelines of the Preferred Reporting Items for Systematic Reviews and Meta-analysis [116]. We searched the PubMed, MEDLINE, EMBASE, PsycINFO, CINAHL, Cochrane Database of Systematic Reviews with the terms Ginkgo* or Gingko* or EGB761 or “EGB 761” or EGB-761. For continuous data, standardized mean differences (SMD) was used. Given the expected heterogeneity, we a priori used a random-effects model. Overall, SMD with 95% CI was estimated with DerSimonian–Laird random-effects models. Publication bias was evaluated by a funnel plot and Egger’s linear regression analysis and *p* < 0.05 was set as the level of significant. This meta-analysis was performed using R 3.6.2.

Papers meting the inclusion criteria were analyzed. Our meta-analysis included a total of eight studies [96,97,109,110,111,112,113,114], encompassing 1120 cases and 1128 healthy controls (Figure 6). As indicated in Figure 6, EGb 761^®^ was significantly effectiveness to placebo as indicated by the dementia scores SKT (SMD = −0.52, 95% CI = −0.99–0.05, *p* = 0.031).

Meta-analysis resulted in a statistically significant difference in favor of EGb 761^®^ therapy respect to placebo (SMD = −0.52, 95% CI = −0.99–0.05, *p* = 0.031).

There was significant heterogeneity I2 = 96.4% (*p* < 0.0001) [115].

Our funnel plot and statistical test showed no evidence of publication bias (Egger’s test z = −0.5069, *p* = 0.612). These analysis supports the notion that EGb 761^®^ is effective in these patients.

EGb 761^®^ treatment has also been studied in patients with psychological and behavioral symptoms of dementia” (BPSD), highlighting an improvement in neuro psychiatric symptoms as well as a clear recovery in behavioral abilities. There is also further evidence of the benefit of the combination of EGb 761^®^ in association with AChEIs in cognitive performance [98,99]. The exact mechanism of action is still unclear. Human pharmacological data show increased potency of EEG in older subjects, a reduction in blood viscosity and improved brain perfusion in specific areas in healthy male subjects (60–70 years) [117,118,119].

In addition, more evidences are needed to assess the role of EGb 761^®^ in combination therapy with AChEIs or memantine or triple, EGb 761^®^, AChEIs and memantine together.

This compound combination, in the future, may have a role in the therapy and in the drug management of dementia, also improving patient rehabilitation. Finally, future investigation will need neuroimaging criteria to better understand the mechanism of action of EGb 761^®^.

## Figures and Tables

**Table 1:** Clinical studies on EGb761EGb 761^®^ in patients with MCI, vMCI, neurocognitive deficit and dementia.

Dementia	Diagnosis	References
Mild cognitive impairment (MCI)	Neuropsychological syndrome characterized by emerging cognitive impairment	[84]
Alzheimer’s disease (AD)	Neurodegenerative disorder characterized by loss of neurons and synapses in the cerebral cortex and certain subcortical regions.	[85]
Vascular dementia (VaD)	Cognitive dysfunctions resulting from brain tissue death due to ischemia caused by vascular disease.	[86]
Frontotemporal lobar degeneration (FTLD)	Neurodegenerative disorders characterized by progressive changes in behavior, personality.	[87]
Dementia with Lewy bodies (DLB)	Several cognitive, behavioral and neurological symptoms characterized by memory loss, hallucinations, rapid eye movement	[88]
Behavioral and psychological and symptoms of dementia” (BPSD)	Neuropsychiatric symptoms and behavioral manifestations (apathy, depression, aggression agitation) associated with dementia.	[89]

## Data Availability

For all data used in the review there is a reference.
